# The trait anger affects conflict inhibition: a Go/Nogo ERP study

**DOI:** 10.3389/fnhum.2014.01076

**Published:** 2015-01-21

**Authors:** Yong Liu, Xianghong Zhan, Wei Li, Heyun Han, Huixia Wang, Junlin Hou, Guoli Yan, Youjie Wang

**Affiliations:** Basic Disciplines of Integrated Traditional Chinese and Western Medicine, School of Basic Medicine, Henan University of Traditional Chinese MedicineZhengzhou, China

**Keywords:** trait anger, conflict inhibition, Go/Nogo, N2, P3

## Abstract

To explore the time course of inhibitory control in high trait anger individuals, we recorded and analyzed ERP data relevant to visual Go/Nogo task in high and low trait anger participants. Compared with low trait anger participants, high trait anger participants revealed faster RTs in the Go/Nogo task. The nogo effect of N2 related to conflict monitoring was similar between two groups. While the P3go was larger in high than low trait anger groups, the P3nogo did not differ between two groups. This induced the smaller nogo effect of P3 in high than that in low trait anger group, which is closely related to the actual inhibition of the motor system. These data suggest the reduced later stage of inhibitory processes in high trait anger individuals, implicating the dysfunction of inhibitory control.

## Introduction

As a common mood in everyday life, anger is considered to be the core mechanism of mood disorders, anxiety and aggression and closely associated with impulsive aggression, damage behavior, etc (Wilkowski and Robinson, 2010). Long-term anger can bring serious impact to the body health and social relations (Deffenbacher et al., 2003; Bettencourt et al., 2006; Shorey et al., 2011). Generally, trait anger is defined “in terms of individual differences in the disposition to perceive a wide range of situations as annoying or frustrating and by the tendency to respond to such situations with elevations in state anger” (Forgays et al., 1997). High levels of trait anger predict quite a few health outcomes (Robinson et al., 2012). For example, angry individuals suffer from excessive cardiovascular reactivity (Suarez et al., 1998), higher blood pressure (Suls et al., 1995), many other physical problems (Williams et al., 2002), and psychological health problems, such as borderline personality (Distel et al., 2012), attempted suicide (Daniel et al., 2009). Especially, angry individuals damage their social relationships (Baron et al., 2007), receive less social support (Smith et al., 2004), and have high divorce rate (Roberts et al., 2007).

Several theoretical perspectives underlying the characteristic trait of angry individuals have been proposed (Wilkowski and Robinson, 2010; Owen, 2011). Recent studies revealed that individuals with superior inhibitory control abilities may be able to override their automatic tendencies toward anger and aggression. For example, Gagne et al assessed the associations between anger and inhibitory control in a twin sample from 12 to 36 months of age and found that twins with low levels of inhibitory control had high levels of anger (Gagne and Goldsmith, 2011). As a core component of inhibitory control, response inhibition is generally defined as the ability to adaptively suppress behavior when environmental contingences demand this (Luijten et al., 2011). Recently, Wilkowski et al. suggested that anger and reactive aggression may represent a more specific aspect of personality and psychopathology directly relevant to response inhibition. Thus, individuals may mobilize the cognitive resources needed for response inhibition when it serves their goals, and they may allow these same resources to lay dormant when they have little motivation to contain inappropriate responses. These findings suggested that low trait anger individuals mobilize these resources when they encounter angry expressions; while high trait anger individuals allow these resources to lie dormant under the same circumstances (Wilkowski, 2012). Recent neuro-imaging research found that trait anger related to increased left frontal cortical activation and that this relationship was not due to anger being regarded as a positive feeling (Pessoa, 2010). In addition, there was evidence that trait anger was inversely associated with the strength of resting-state functional connectivity (RSFC) between the amygdala and the contralateral middle orbitofrontal cortex, especially for the right amygdale- left orbitofrontal connectivity (Fulwiler et al., 2012). To this end, previous findings showed that the difference of the inhibitory control might be the foundation of trait anger; however, the time course of this inhibitory control had never been reported for trait anger, which will be investigated by recording and analyzing ERPs in response to Go/Nogo task relevant to inhibitory control.

The Go/Nogo paradigm is widely used for the assessment of inhibitory control (Pfefferbaum et al., 1985). Two ERP components with a frontal-central distribution, N2nogo and P3nogo, are enhanced in response to Nogo trails over Go trials, reflecting changes in brain activity related to response inhibition (Yang et al., 2009). The N2nogo is a negative wave that emerges approximately 200–300 ms after stimulus presentation and the P3nogo is a positive wave that emerges 300–600 ms after stimulus onset. Geczy et al. suggested that increased N2 amplitude in response to Nogo stimuli after Go cues might be related to increased efforts to activate the response inhibition system and to interrupt preparations for response execution (Géczy et al., 1999). Converging evidence suggested that the N2nogo amplitude is a valuable measure for response inhibition. In contrast, Nieuwenhuis suggested that the N2nogo reflects response conflict rather than inhibition because it was enhanced for low-frequency stimuli and was localized to the anterior cingulated cortex (Nieuwenhuis et al., 2003). Although the overlapping of movement-related activities may influence the difference between Go and Nogo ERPs within this time range, the P3nogo is generally related to the later stage of the inhibition process that is closely related to the actual inhibition of the motor system in the premotor cortex (Bokura et al., 2001; Kok et al., 2004; Smith et al., 2008; Verleger et al., 2009). In addition, it has been suggested that the difference waves (Nogo- minus Go-ERPs) would reflect the Go/Nogo effect and further specifically reflect frontal inhibitory functioning, that is the difference waves of N2 (N2d) and P3 (P3d), which were defined as the results of N2go subtracted from the N2nogo and P3go subtracted from the P3nogo, respectively (Bokura et al., 2001). Although there are still many controversial reports, it was widely accepted that these components elicited by Nogo stimuli, i.e., N2nogo (N2d) and P3nogo (P3d), are associated with two aspects of inhibitory control, i.e., conflict monitoring and response inhibition (Yang et al., 2009). To date, few studies investigated response inhibition in high and low trait anger by use of ERPs, which will be conducted in the present visual Go/Nogo paradigm.

## Methods

### Participants

Two thousand six hundred and forty two participants (1533 female; 25.3 ± 4.8 years) completed the STAXI-2 (State-Trait Anger Expression Inventory 2)^1^ and the handedness scale (Spielberger, 1988). Trait anger was assessed by the trait anger scale (TAS)^2^ and the TAS score for all participants was 17.81 ± 3.75. According the TAS scores of each participant, we selected those subjects with higher TAS score for the high anger trait group (TAS score ≥ 22) and lower TAS score for the low anger trait group (TAS score ≤ 14). To address the present question, then, we selected randomly 16 participants (eight female; 24.3 ± 2.8 years) from the high anger trait group and 16 participants from the low anger trait group (eight female; 24.1 ± 3.4 years), respectively. All of 32 participants were right handed and free of medication for at least 24 h before testing, with normal or corrected-to-normal vision, without history of head trauma or other medical conditions that could cause cognitive impairment (Yang et al., 2009). The study was conducted in accordance with the Declaration of Helsinki and all procedures were carried out with the adequate understanding and written informed consent of the subjects. The study protocol was approved by the Ethics Committee of the Institute of Psychology of the HACTCM.

### Stimuli and procedure

Visual stimuli included single and double triangles in gray background, presented in the center of a computer screen (light degree = 60 cd/m^2^). Participants were seated in a semi-dark room, facing a monitor place 75 cm from their eyes, with a visual angle of 4° × 4°. There were four blocks with 60 Go (double triangles) and 40 Nogo (single triangle) stimuli for each. The participants were instructed to respond by pressing a button as quickly as possible after the Go stimuli appeared and to withhold the response when the Nogo stimuli appeared. Each stimulus was presented for 100 ms, with the mean inter-stimulus intervals (ISI) being 1200 ms (randomly between 1000 and 1400 ms). The hand to press button was counterbalanced across the participants. Before EEG recording, participants performed one practice block consisting of 40 Go and Nogo trials. During the experiment, participants were instructed to watch the center of the screen, relax, and minimize eye blinks or body movements.

### EEG recording and analysis

Based on the present aim, EEG signals were continuously recorded (band pass 0.05–100 Hz, sampling rate 500 Hz) using 32-channel Ag/AgCl electrodes cap (10–20 International System; Quick Cap, www.neuroscan.com) by NuAmps amplifier, referenced to the left mastoid (right mastoid as recording site). VEOG and HEOG were recorded with two pairs of electrodes, one placed above and below right eye, and the other 10 mm from the lateral canthi. Electrode impedance was maintained below 5 kΩ throughout the experiment.

We used EMSE 5.5 software (www.sourcesignal.com) to analyze the data off-line. EEG data were re-referenced to the bi-mastoid average reference. EOG artifacts were corrected offline. The EEG was segmented into the epoch from 200 ms pre-stimulus to 1000 ms post-stimulus. Trials contaminated by amplifier clipping, bursts of electromyographic activity, or peak-to-peak deflection exceeding ±100μv were excluded from averaging. The EEG segments were averaged separately for target and standard stimuli. The number of average trials left after removal of the artifacts was 130 (Nogo) and 202 (Go) for low and 125 (Nogo) and 208 (Go) for high trait anger, respectively.

Behavioral results (RT and accuracy) were compared by *t*-test to explore the group difference between high and low trait anger groups. For ERPs data, according to the frontal-central scalp distribution of N2nogo and P3nogo components, we focused on the analysis at frontal-central electrode sites (Fz, FCz, Cz). The N2 component was quantified as the most negative amplitude within a 200 to 300 ms window following stimulus onset. The P3 component was quantified as the most positive amplitude within 300 to 600 ms following the N2 peak. In order to highlight the Nogo effect, difference waves (N2d and P3d; Nogo- minus Go-ERPs) were computed (Yang et al., 2009). The measurements of peak latencies and amplitudes of N2 and P3 components were subjected to Three-Way repeated measures ANOVA with Stimulus (Go, Nogo) and Site (Fz, FCz, Cz) as within-subject factors and Group (high, low) as between-subject factor. A Site (Fz, FCz, Cz) × Group (high, low) repeated measures ANOVA was performed for the measurements of N2d and P3d components. The Geisser–Greenhouse correction was used for any repeated measures containing more than one degree of freedom in the numerator (Geisser and Greenhouse, 1958).

## Results

### Behavioral data

Behavioral data indicated that RTs was faster in the high score group (302 ms) than that in the low score group (328 ms; *t* = 2.54, *p* = 0.017). The accuracy did not differ between two groups (96.3 and 95.6% for high and low score groups, respectively; *t* = 0.66, *p* = 0.51).

### ERPs data

**Figure 1:**
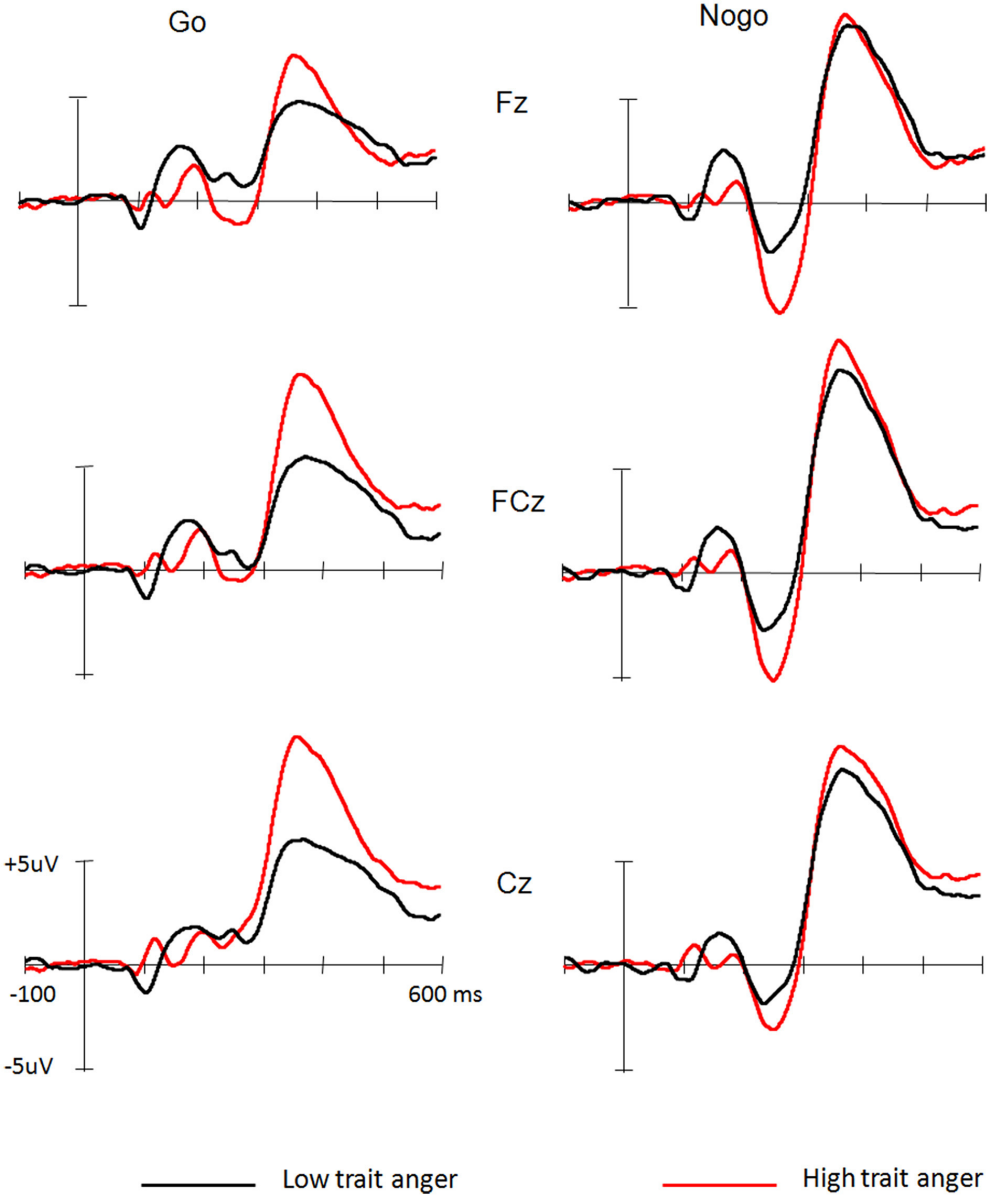
**Grand average waveforms elicited by Go and Nogo stimuli in low and high trait anger groups, respectively**.

Figure 1 illustrates the grand averages for the Go and Nogo stimuli in the high and low trait anger groups, respectively. Compared with the ERP waveforms elicited by Go stimuli, Nogo stimuli elicited larger frontal-central N2 (i.e., N2nogo) and P3 (i.e., P3nogo) components, regardless of high or low score group.

Because across Go and Nogo stimuli we did not find any significant group differences for latencies of N2 [*F*_(1, 26)_ = 0.69, *p* = 0.41] and P3 [*F*_(1, 26)_ = 0.15, *p* = 0.69] components, the following analysis focused on the amplitude of each component.

The N2 was slightly larger for low (−4.5 μV) than high [−2.7 μV; *F*_(1, 26)_ = 0.99, *p* = 0.33] trait anger groups. Across participants' groups, the N2 amplitudes were overall larger for Nogo (−5.2 μV) than for Go stimuli [-2.0 μV; *F*_(1, 26)_ = 12.89, *p* = 0.005]. The main effect for Site was also significant, *F*_(2, 52)_ = 10.81, *p* < 0.001, with the largest N2 (−4.1 μV) at FCz site. No interactions were significant (*p*s > 0.1).

Similar to the analysis of N2 component, the P3 amplitudes were overall larger for Nogo (−5.2 μV) than for Go stimuli [−2.0 μV; *F*_(1, 26)_ = 12.90, *p* = 0.004]. The main effect for Site was also significant, *F*_(2, 52)_ = 9.88, *p* < 0.001, with the largest P3 (10.4 μV) at Cz site. Importantly, a Group × Stimulus interaction was found, *F*_(1, 26)_ = 4.34, *p* = 0.041. *Post-hoc* analysis indicated that the P3 elicited by Go stimuli was significantly reduced in low (7.2 μV) over high score group [9.9 μV; *F*_(1, 26)_ = 5.06, *p* = 0.033], whereas the P3nogo did not differ between two groups [10.4 and 11.4 μV for low and high score groups, respectively; *F*_(1, 26)_ = 0.41, *p* = 0.53], and that, while the nogo effect of P3 component was significant in low score group [*F*_(1, 26)_ = 16.10, *p* < 0.001], it was not evident in high score group [*F*_(1, 26)_ = 1.14, *p* = 0.29].

**Figure 2:**
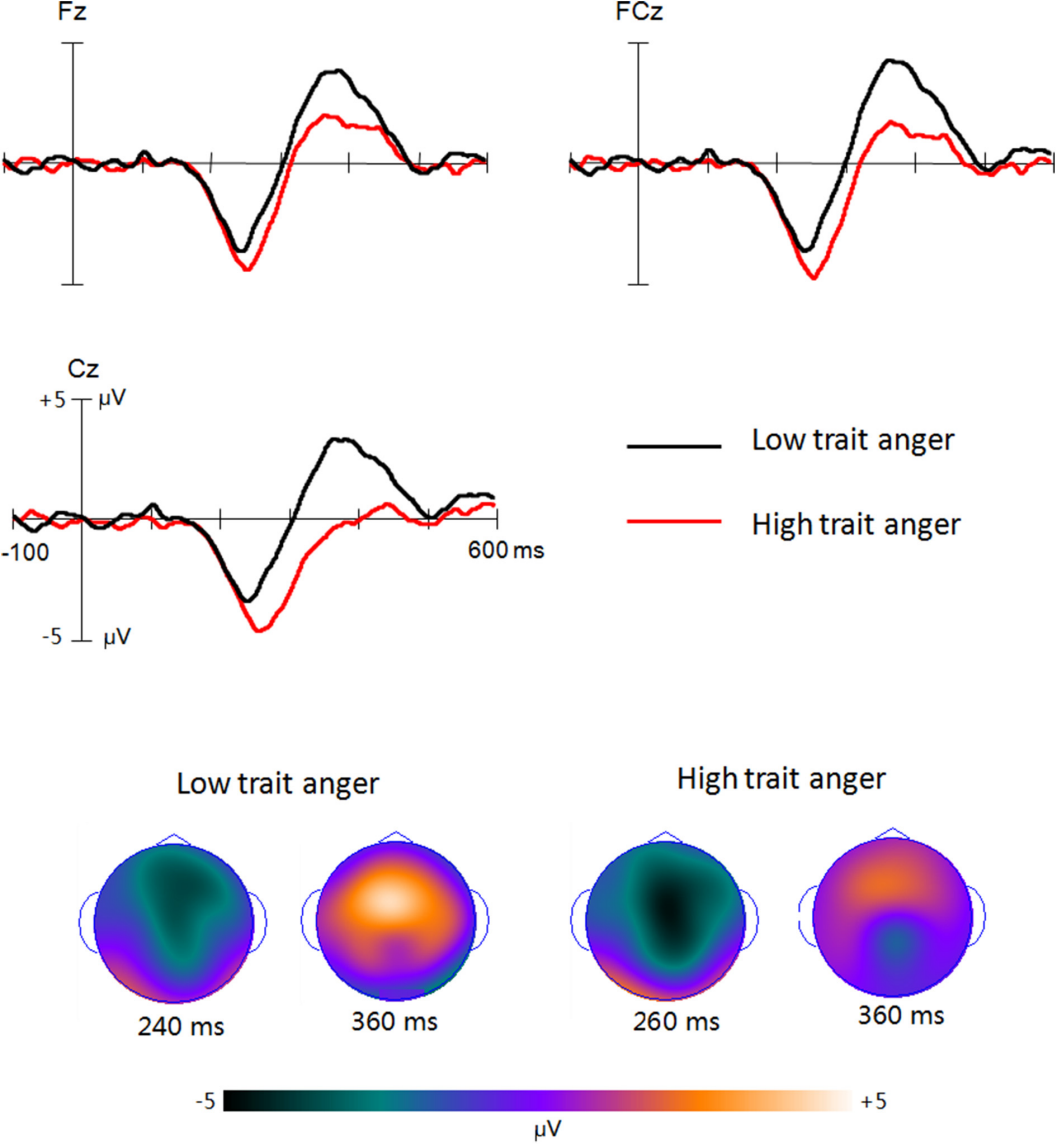
**The difference waveforms between Nogo and Go ERPs in low and high trait anger groups, respectively, as well as the 2D scalp distribution topography of the peak amplitudes of N2 and P3 components**.

For the analysis of difference waveforms related to inhibition effects (Figure 2), the ANOVA revealed that the amplitude of N2d was similar between two groups [−2.5 and −3.3 μV for high and low trait anger groups, respectively; *F*_(1, 26)_ = 2.42, *p* = 0.132], whereas the P3d was significantly reduced in high (0.4 μV) than low (2.2 μV) trait anger group [*F*_(1, 26)_ = 6.83, *p* = 0.024].

## Discussion

The main aim of the present study was to investigate the effects of the trait anger on response inhibition. Compared with low trait anger participants, high trait anger participants revealed faster RTs in the Go/Nogo task. Across groups, the nogo stimuli elicited larger N2 and P3 components than did the go stimuli. Although the nogo effect of N2 was similar between low and high trait anger groups, the P3 elicited by Go stimuli was larger in high than low trait anger groups and the P3nogo did not differ between two groups. This induced smaller P3d component in high over low trait anger groups.

Consistent with the present RT data, Parrott et al reported that high levels of trait anger displayed facilitative biases in the processing of semantic anger-related stimuli (Parrott et al., 2005). However, this effect was not found widely (Wenzel and Lystad, 2005). Indeed, in previous studies the stimuli with anger information were presented to participants and the state anger after the experiment was not measured; hence, it is hard to contribute the change of state anger and/or trait anger to the RT difference between participants with low and high levels of trait anger (Wenzel and Lystad, 2005). The present experiment presented a simple triangle shape without any emotional information, which would not change the state anger of participants. The fast performance in participants with high level of trait anger is indeed consistent with the idea that anger is associated with greater impulsiveness (Ramírez and Andreu, 2006; Jaworska et al., 2012).

Consistent with previous studies, greater N2 amplitudes were observed in Nogo than in Go trials, regardless of the trait anger. There was evidence that the N2nogo amplitude is a valuable indicator for the measurement of response conflict. The present fact that the N2nogo as well as N2d did not differ between high and low angry persons indicated that the response conflict especially the detection of conflict information is not modulated by the trait anger.

Although there was evidence that the P3nogo component may not necessarily represent inhibition of a response (Falkenstein et al., 1999; Fallgatter and Strik, 1999), it is accepted that the P3nogo is related to the outcome of the inhibition process and reflects the conflict inhibition processing (Overtoom et al., 2002; Dimoska et al., 2003). The reduced P3d amplitudes in the present high trait anger group indicated the impaired response inhibition. Supporting this view, one recent report showed that the anger group had more false alarms overall, indicating impaired response inhibition (Jaworska et al., 2012). In addition, the increased right cortical activation during the initial portion of CPT existed in the anger group, perhaps reflecting greater engagement of frontal circuits (i.e., effort) during initial stages of the task compared to controls, revealing a hyper-vigilant state in anger group, which may interfere with effective attention control and decrease inhibition (Jaworska et al., 2012).

It should be noted here that the reduced Pd3 in high trait anger group is indeed due to the decreased P3go in low trait anger group. In line with recent reports (Shucard et al., 2008), the present findings implicated that the hyper-arousal reflected by higher amplitude of target P3 might be a common feature of high trait anger participants. Generally, the hyper-arousal plays the primary role in producing an enhanced P3 to Go stimuli, and the high score individuals cannot mobilize more inhibitory resources on Nogo stimuli. Our behavioral findings of faster response to Go stimuli in the high trait anger group supported this hypothesis as well. It is reasonable that, in the cognitive processes from stimuli to response, high trait anger individuals have an inferior inhibitory control and then make a quick emotional response.

In sum, the present study explored the time course of inhibitory control in high trait anger individuals by recording and analyzing ERP data relevant to visual Go/Nogo task. While, the nogo effect of N2 related to conflict monitoring was similar between two groups, the nogo effect of P3 closely related to the actual inhibition of the motor system was smaller in high than that in low trait anger group. These data suggest the reduced later stage of inhibitory processes in high trait anger individuals, implicating the dysfunction of inhibitory control.

### Conflict of interest statement

The authors declare that the research was conducted in the absence of any commercial or financial relationships that could be construed as a potential conflict of interest.

## Footnotes

^1^STAXI – State-Trait Anger Expression Inventory is a 44-item questionnaire and is extensively utilized in research on anger. It distinguishes between the three modes of anger expression: anger-out, anger-in, and anger-control. Anger-out refers to a tendency to express anger through either verbal or physical behaviors. Anger-in or suppressed anger refers to the tendency to hold one's anger on the inside without any outlet. Anger-control refers to the tendency to engage in behaviors intended to reduce overt anger expression (Spielberger, 1988).

^2^Generally, State-Trait Anger Scale (STAS) includes 10 items and is built upon two subscales: State anger, defined as an emotional state or condition that consists of subjective feelings of tension, annoyance, irritation, fury and rage; Trait anger defined in terms of individual differences in the frequency that State anger was experienced over time. We recorded the trait anger scale in the present study. (Spielberger et al., 1983)
